# Intracellular Water Content in Lean Mass as an Indicator of Muscle Quality in an Older Obese Population

**DOI:** 10.3390/jcm9051580

**Published:** 2020-05-22

**Authors:** Mateu Serra-Prat, Isabel Lorenzo, Mònica Papiol, Elisabet Palomera, Maria Bartolomé, Eulogio Pleguezuelos, Emili Burdoy

**Affiliations:** 1 Research Unit, Consorci Sanitari del Maresme, Mataró, 08304 Barcelona, Spain; ilorenzo@csdm.cat (I.L.); epalomera@csdm.cat (E.P.); 2 Institut de Recerca Germans Trias i Pujol (IGTP), Badalona, 08916 Barcelona, Spain; 3 Centro de Investigación Biomédica en Red de Enfermedades Hepáticas y Digestivas (CIBEREHD), ISCIII, 08304 Barcelona, Spain; 4 Universitat de Vic-Universitat Central de Catalunya, 08500 Barcelona, Spain; 5 ABS Argentona, Consorci Sanitari del Maresme, Argentona, 08310 Barcelona, Spain; mpapiol@csdm.cat (M.P.); eburdoy@csdm.cat (E.B.); 6 ABS Mataró Centre, Consorci Sanitari del Maresme, Mataró, 08304 Barcelona, Spain; mbartolome@csdm.cat; 7 Rehabilitation Service, Hospital de Mataró, Consorci Sanitari del Maresme, Mataró, 08304 Barcelona, Spain; epleguezuelos@csdm.cat

**Keywords:** intracellular water, muscle quality, strength, sarcopenia, balance, gait, obese, aged

## Abstract

Background: In aged populations, muscle strength depends more on muscle quality than on muscle quantity, while all three are criteria for the diagnosis of sarcopenia. Intracellular water content (ICW) in lean mass (LM) has been proposed as an indicator of muscle quality related to muscle strength in older people. Objectives: To evaluate the relationship between the ICW/LM ratio, muscle strength and indicators of functional performance in obese older adults, and to assess the value of the ICW/LM ratio as an indicator of muscle quality. Methodology: Design: cross-sectional study. Population: persons aged 65–75 years with a body mass index of 30–39 kg/m^2^. ICW and LM were estimated by bioelectrical impedance. Hand grip, gait speed, unipedal stance test, timed up-and-go (TUG) test, Barthel score and frailty (Fried criteria) were assessed. Sarcopenia was established according to EWGSOP2 criteria. Results: Recruited were 305 subjects (66% women), mean age 68 years. The ICW/LM ratio correlated with the TUG test, gait speed and grip strength, and was also associated with sex, the unipedal stance test and frailty. Independently of age, sex and muscle mass, the ICW/LM ratio was related with gait speed, the TUG test and unipedal stance capacity. One person (0.3%) had sarcopenia defined as low muscle strength and low muscle mass, while 25 people (8.2%) had sarcopenia defined as low muscle strength and poor muscle quality (ICW/LM). With this last definition, sarcopenia was related to frailty, gait speed and the TUG test. Conclusions: ICW content in LM could be a useful muscle quality indicator for defining sarcopenia. However, more studies are required to confirm our findings for other populations.

## 1. Introduction

Age-related loss of muscle strength is a main determinant of functional capacity in the elderly population and is also closely related to frailty, disability and mortality [1,2,3,4]. It is well known that body composition undergoes several changes with age; one such change is a progressive decrease in skeletal muscle mass (MM) with age from adulthood [5]. Loss of MM has been related to loss in muscle strength and it was previously thought that this loss was a direct consequence of a reduction in muscle size. However, different studies have shown that this relationship is not so clear-cut and that loss in strength with age is only partially explained by loss of MM [6]. A longitudinal study has shown that muscle strength decline is much more rapid and pronounced than MM decline and that changes in the quadriceps area only explain 6%–8% of a loss in knee extensor strength [7]. Other studies have reported similar results, indicating that loss of MM explains less than 10% of the associated loss in muscle strength [8], and that maintaining or increasing MM does not necessarily prevent strength loss [9]. All this evidence suggests that muscle weakness in aged populations is related to factors other than muscle size; for instance, an impaired intrinsic force-generating capacity of the muscle tissue, which can be generally considered as impaired muscle quality [6]. In 2018, the European Working Group on Sarcopenia in Older People (EWGSOP) updated its definition of sarcopenia to include more recent evidence in a new EWGSOP2 (2018) definition of sarcopenia that considers (a) that the main parameter of sarcopenia is low muscle strength, (b) that sarcopenia is possible when low muscle strength is detected, and (c) that sarcopenia is confirmed when low muscle strength is accompanied by low muscle quantity or quality. While the criteria for assessing muscle strength and muscle quantity are well established, the EWGSOP, recognizing the limitations on assessing muscle quality, encourages the development of new evaluation instruments and methods in the near future [10].

As a person ages, the quantity of total body water (TBW) and intracellular water (ICW) also decreases [11]. Muscle represents 40%–50% of total body weight in the aged population and approximately 76% of muscle content is water. Cell volume plays a critical role in mediating insulin effects in different mammalian cell types. The mechanisms that regulate cell water balance are therefore important for skeletal muscle, a major site of insulin action [12]. Dehydration affecting the muscle has important effects on both the mechanical and metabolic functions of muscle. Muscle is responsible for most glucose metabolism and plays a major role in the development of insulin resistance and in the treatment of type 2 diabetes mellitus (for which one of the most effective treatments is physical exercise). Insulin promotes glucose uptake, mainly by the liver and muscle cells. It has been suggested that glycogen and water recovery in muscle after exercise is a coordinated process [13], although more studies are needed to confirm this finding.

An age-related decrease in ICW may reflect a loss in the number of muscle cells (i.e., in MM) but may also reflect a reduction in muscle cell hydration. It has been reported that higher levels of ICW are associated with better functional performance and a lower frailty risk in community-dwelling aged populations [14]. However, that same study did not clearly elucidate whether high ICW content was due to greater MM or to better muscle cell hydration. ICW content in lean mass (LM), i.e., the ICW/LM ratio (in mL/kg), as measured by bioelectrical impedance analysis (BIA), has been proposed as an indicator of muscle quality and cell hydration [15]. In a cross-sectional study, this ratio was related to greater muscle strength, better functional capacity and a lower frailty risk, independently of age, sex, comorbidities and LM [14], suggesting that the ICW content in LM may play a relevant role in muscle function.

The aim of this study was to assess the relationship between the ICW/LM ratio and muscle strength, functional capacity and different indicators of balance and gait, as well as to explore the value of the ICW/LM ratio as an indicator of muscle quality in the definition of sarcopenia in obese community-dwelling adults aged 65–75 years. The opportunity was also taken to validate the BIA as a relatively easy-to-use measurement method for routine clinical practice in primary care centres.

## 2. Methodology

### 2.1. Study Design and Population

This observational cross-sectional study is nested in a randomized controlled trial (PRE-FROB study, registered under ID NCT03000907 in ClinicalTrials.gov PRS) aimed at assessing the effectiveness of a multimodal intervention in community-dwelling obese older adults. Data used for this analysis come from baseline assessments of participants before initiating any intervention.

Participants were aged 65–75 years, had a body mass index (BMI) of 30–39 kg/m^2^ and had at least one of the following obesity-related clinical conditions: dyslipidaemia, hypertension, diabetes or insulin resistance, obesity-related physical limitations and sleep apnoea/hypopnea. Exclusion criteria were dementia, neurodegenerative diseases, severe psychiatric disorders, cancer diagnoses, life expectancy of <6 months and institutionalization. Recruitment took place in 3 primary care centres in Mataró (Spain) from February to June 2017. All eligible subjects who fulfilled inclusion criteria were invited to participate and those who agreed to participate gave their written informed consent before recruitment. The study protocol was approved by the local research ethics committee (CSdM CEIC reference number 60/16).

### 2.2. Data Collection

Data was collected strictly following the BIA and Bodygram software guidelines (Akern SRL, Firenze, Italy. EFG3 50-Hz BIA device; Bodygram 3.1 software package).

Participants received guidelines on typical physical activity, diet and hydration for the measurement day and the preceding preparation day. For the preparation day, participants were instructed not to consume any alcoholic beverage or caffeine (including coffee) and to avoid excessive physical activity; for the measurement day, when measurements had to be made in a fasting state, participants were instructed not to eat or drink anything in the 4 h before measurement, to urinate 30 min before measurement and to defecate before measurement. BIA was performed in standard conditions and after ensuring that the individual did not show evident clinical signs or symptoms of dehydration. The standard Bodygram software data entry form was completed for each participant with details of their age, sex, height and weight.

Subjects, in light clothing without jewellery or metal elements on their body, were asked to remain in a horizontal position for 10 min before measurement. Measurement was in the supine recumbent position, with arms and legs separated from the trunk by about 30° and 45°, respectively (as recommended by the manufacturer). Four electrodes were connected on the same side of the body, the first pair to the dorsal region of the hand and the second pair to the dorsal region of the foot. Electrical current was applied to the source (distal) electrodes and the fall in tension due to impedance was detected by the proximal electrodes. The BIA resistance and reactance measurements were interpreted by the Bodygram software algorithms, with the following data furnished in the corresponding report: LM (in kg), MM (in kg), TBW (in mL), extracellular water (ECW, in mL), and ICW (in mL, calculated as the difference between TBW and ECW). The ICW/LM ratio (in mL/kg) is used as an indicator of muscle quality.

Used as a measure of muscle strength was hand-grip strength (in kg) in the dominant hand, measured using the hand-held JAMAR dynamometer. Balance was assessed by the unipedal stance test, which measures whether a person is capable of standing on one foot for more than 5 s. The number of falls in the previous 3 months was recorded. Measured gait speed was used as an indicator of walking ability. Anthropometric measurements (weight, height, BMI) were recorded. Functional performance was assessed by the Barthel score and the timed up-and-go (TUG) test. Frailty status was established according to 5 Fried criteria [16], namely, weight loss, exhaustion, poor physical activity, slow gait speed and weakness, and persons were classified as robust, pre-frail and frail if they fulfilled 0, 1–2 and 3+ criteria, respectively. Sarcopenia was defined according to the EWGSOP2 criteria [10] and an ICW/LM ratio <1st quintile was considered a criterion for low muscle quality.

Other study variables included sociodemographic characteristics, comorbidities, number of chronic medications, nutritional status assessed using the short-form Mini Nutritional Assessment (MNA-sf) and certain biochemical blood indicators of inflammation, namely, C-reactive protein (CRP), interleukin-6 (IL-6) and glycaemic control (glycaemia, HbAc1), as determined by validated commercial kits.

### 2.3. Statistical Analysis

Continuous variables were described using means and standard deviation (SD) and categorical variables were described using percentages. The ICW/LM ratio was correlated with other continuous variables using the Spearman correlation coefficient (rs). The Mann–Whitney U test was used to compare the ICW/LM ratio between two groups. Linear regression analysis was used to assess the relationship of the ICW/LM ratio with other continuous variables (gait speed, TUG test, hand grip), while logistic regression analysis was used to assess the relationship of the ICW/LM ratio with dichotomous variables (unipedal stance capacity, robustness). Multivariate linear and logistic regression analyses were used to adjust the effect of the ICW/LM ratio on main outcome measures for age, sex and MM. Regarding frailty status, robustness (robust vs. pre-frail and frail) was considered instead of frailty (frail vs. pre-frail and robust) because of the very small number of frail cases in our sample. All analyses were performed for the overall sample and for men and women separately to assess interactions according to sex. The ICW/LM ratio was separately observed for men and women. A 20th percentile criterion was finally adopted to properly discriminate patients with low ICW/LM ratios. The 10th percentile criterion was ruled out to reduce the risk of statistical anomalies arising from using a small sample dataset. To assess the value of the ICW/LM ratio as an indicator of muscle quality in the definition of sarcopenia, the relationships between sarcopenia and gait speed and the TUG test were evaluated using the Mann–Whitney U test and the relationships between sarcopenia and frailty, falls and the unipedal stance test were evaluated using the chi-squared test. Statistical significance was set to *p* < 0.05.

## 3. Results

A total of 305 subjects were recruited (65.9% women). Mean age was 69.7 (2.7) years and mean BMI was 34.1 (3.3). The main comorbidities were arterial hypertension (76.1%), dyslipidaemia (66.9%), diabetes (26.2%), gastroesophageal reflux (24.6%), peripheral vasculopathy (23.9%) and depression (22.6%). The individuals in the sample had a mean of 3.5 (1.5) comorbidities, were taking a mean of 4.9 (83.7) medications and scored very well on functional capacity (mean Barthel score 99.2), while 94.1% had a good nutritional status (MNA-sf > 11). Body composition and functional characteristics of the study sample are presented in Table 1.

The mean (SD) ICW/LM ratio was 426 (50) mL/kg for the overall sample, but significant differences were observed in mean (SD) values for men and women (435 (49) mL/kg vs. 421 (51) mL/kg; *p* = 0.001). The ICW/LM ratio was not related to age; the non-correlation of the ICW/LM ratio with age is possibly explained by the relatively narrow variance observed (2.7). While the ICW/LM ratio was not related to comorbidities other than diabetes (415 mL/kg for subjects with diabetes vs. 427 mL/kg for subjects without diabetes; *p* = 0.005), this ratio was negatively correlated with the comorbidity load (expressed as the number of comorbidities and number of medications), as shown in Table 2.

In relation to robustness, the ICW/LM ratio was 433 (57) mL/kg in robust subjects, 423 (45) mL/kg in pre-frail subjects and 386 (43) mL/kg in frail subjects (*p* = 0.020). For men, the corresponding figures were 442.8 (45.7) mL/kg in robust subjects and 424.9 (51.7) mL/kg in pre-frail subjects (*p* = 0.057); there were no frail men. The corresponding figures for women were 422.8 (66.6) mL/kg in robust subjects, 422.3 (43) mL/kg in pre-frail subjects and 386.0 (42.8) mL/kg in frail subjects (*p* = 0.129).

Regarding the unipedal stance test, the ICW/LM ratio was higher in subjects who passed (429 (51) mL/kg) than in subjects who failed (406 (37) mL/kg) (*p* = 0.001). As for falls, the ICW/LM ratio was lower in fallers than in non-fallers (415.6 (54) mL/kg vs. 427.4 (50); *p* = 0.053). Crude relationships between the ICW/LM ratio and different numeric indicators of functionality are presented in Table 2.

Overall, the bivariate analysis showed that the ICW/LM ratio was associated with sex, the TUG test, gait speed, hand grip, unipedal stance test and frailty status and, furthermore, when adjusted for age, sex and MM, those relationships were statistically significant for the TUG test, gait speed and the unipedal stance test. Multivariate models did not point to any significant independent association of the ICW/LM ratio with hand grip or robustness (see Table 3 and Table 4).

**Figure 1:**
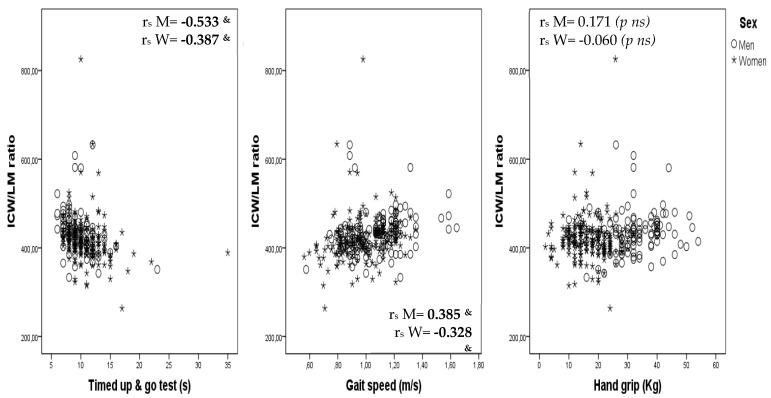
Correlations of intracellular water/lean mass (ICW/LM) ratio and timed up-and-go test, gait speed and hand grip, by sex. rs M: Spearman correlation in men. rs W: Spearman correlation in women (*p* < 0.001).

Correlations between ICW/LM ratio and the TUG test, gait speed and hand grip are shown, by sex, as scatterplots in Figure 1.

One person (0.3%) had sarcopenia defined as low muscle strength and low muscle mass; this person also had poor muscle quality (ICW/LM <1st quintile). Under a tenth of people (*n* = 25; 8.2%) had sarcopenia defined as low muscle strength and poor muscle quality (5.8% men and 9.5% women; *p* = 0.266). Sarcopenia was related with frailty (with a prevalence of 0.8% in robust individuals, 11.8% in pre-frail individuals and 50.0% in frail individuals; *p* < 0.001), gait speed (1.02 m/s in non-sarcopenic individuals vs. 0.89 m/s in sarcopenic individuals; *p* = 0.001), the TUG test (10.0 s in non-sarcopenic individuals vs. 12.6 s in sarcopenic individuals; *p* < 0.001) and the 2-min walking test (133.7 m in non-sarcopenic individuals vs. 116.1 m in sarcopenic individuals; *p* < 0.001), but did not reach statistical significance for the Barthel score (*p* = 0.093) or the unipedal stance test (*p* = 0.123). Table 5 shows that the ICW/LM ratio along with low muscle strength was associated, in a logical and plausible way, with greater frailty, more falls and poorer TUG test, gait speed and unipedal stance test results. These findings would validate the usefulness of the proposed muscle quality indicator. Evidently, further studies with larger samples and broader age ranges are necessary to corroborate these tentative findings.

## 4. Discussion

This study shows that the ICW/LM ratio is related to gait speed, balance (the ability to stand on one foot for more than 5 s) and functional performance (assessed by the TUG test) in obese community-dwelling subjects aged 65–75 years. However, no independent relationship of the ICW/LM ratio with hand grip and robustness was observed after adjustment for age, sex and MM. Moreover, the results obtained also show that: (a) the use of the ICW/LM ratio as an indicator of muscle quality in the definition of sarcopenia makes it possible to identify a higher number of people with sarcopenia, (b) sarcopenia confirmed by the ICW/LM criteria is related in a logical and plausible way with frailty and physical performance, and (c) considering both loss of muscle quality and low muscle strength better identifies aged people with functional impairment.

A recent study that proposed the ICW/LM ratio as an indicator of muscle quality demonstrated its independent association with hand grip, frailty and functional capacity in community-dwelling elderly people, suggesting that ICW content in LM may play a key role in muscle function and physical performance [15]. In this study we aimed to corroborate those previously observed results for a different sample, consisting specifically of obese adults aged 65–75 years. Although the observed results show that the ICW/LM ratio is independently related to gait speed, the TUG test and the unipedal stance test, the study failed to corroborate an independent association with muscle strength and frailty. The discrepancies may be due to between-study differences in certain characteristics; subjects in our study compared to those in previously published studies [14,15] were 10 years younger on average, had a better functional capacity (Barthel score 99 vs. 96) and a lower prevalence of frailty (2% vs. 14%), while men (but not women) had greater muscle strength (33 vs. 31 kg).

The better and more homogeneous muscular and functional characteristics of the individuals in our study limits our capacity to identify associations between the ICW/LM ratio and functional performance indicators. Nonetheless, the lack of a relationship between the ICW/LM ratio and muscle strength is somewhat contradictory of the finding of an independent relationship with gait speed, the TUG test and the unipedal stance test, since it is thought that gait speed and balance are mainly determined by muscle strength. This apparently contradictory result is possibly explained by the fact that walking and standing on one foot are mainly determined by a large number of lower limb muscles, while muscle strength (assessed by hand grip) is mainly determined by a small number of upper limb muscles, and it is possible that ICW loss does not affect lower and upper limbs in the same way. Some evidence suggests that training may stimulate intracellular muscle hydration [17], so different muscle training packages may cause intracellular hydration to vary between muscles. New studies assessing specific muscle hydration and function packages are needed to elucidate the role of muscle cell hydration on muscle strength and functionality. Regarding frailty status, the lack of an independent association with the ICW/LM ratio may be due to the small number of frail cases in our study sample, clearly limiting the study’s statistical power, especially when stratified by sex.

While TBW and ICW decrease with age, water content in LM is relatively constant over time and so is not affected in healthy ageing populations. Nonetheless, ICW content in LM can vary slightly depending on certain diseases and clinical conditions. Yamada and associates [18] used the ECW/ICW ratio, measured by BIA, as an indicator of muscle quality, demonstrating a significant relationship with muscle strength and gait speed in elderly subjects. There is increasing evidence suggesting that the amount of ICW relative to ECW, TBW and LM may be an indicator of muscle quality and cell hydration and so be related to muscle strength and physical performance [14,15,18].

Ageing is characterized by a progressive process of dehydration that parallels a progressive decline in muscle strength. The relationship between these phenomena is not well understood, but a recent review has hypothesized that age-related hyperosmotic stress and cell dehydration could be major contributors to age-related muscle strength loss, frailty and functional decline [19]. The fact that age impairs the thirst sensation and the capacity to concentrate urine [20] favours low-grade chronic dehydration and hyperosmotic stress, leading to intracellular dehydration (cell shrinkage), an important inflammatory response, increased reactive oxygen species production and certain metabolic and cardiovascular disorders [21,22]. Ageing also causes a reduction in MM associated with ICW loss. Since the main components of LM are TBW and MM, the ICW/LM ratio reflects the level of intracellular hydration of LM and thus of the MM as well, regardless of the changes occurring in the MM. There is evidence that cell volume, mainly determined by ICW, is a metabolic signal that regulates cell function, with cell swelling stimulating anabolism and cell shrinkage stimulating catabolism and protein degradation [23]. It has also been observed that cell dehydration inhibits the mammalian target of the rapamycin (m-TOR) pathway [24] and the metabolic pathways stimulated by insulin [25], leading to anabolic resistance and insulin resistance and affecting muscle growth, muscle regeneration and protein synthesis [26]. The mechanisms underlying these effects are not well understood, but it has been suggested that they may result from impaired protein folding, given that proteins acquire their functional structure through a folding phenomenon mainly driven by hydrophobic interactions [27]. Moreover, muscle dehydration also affects contractile capacity [28] and, although the causes of this impaired contractile capacity are not fully understood, they have been related to water bonds in contractile proteins [29]. While the role of cell hydration in muscle function in aged populations has been poorly investigated and requires further studies to assess both cell hydration and muscle function in the same muscle packages, the existing evidence suggests a key role for hydration in muscle function in aged populations and points to the importance of early detection and correction of chronic low-grade dehydration in that population.

In relation to the updated definition of sarcopenia proposed by EWGSOP2, introduced is the criterion of “muscle quality” as a relatively new term that refers to micro- and macroscopic changes in muscle composition [10]. Although muscle fat infiltration (assessed by imaging tools), the muscle strength/mass ratio and the phase angle assessed by BIA have all been proposed as indicators of muscle quality, there is no consensus on those assessment methods, for which reason the EWGSOP has called for the development and proposal of new indicators [10]. In a recent study of a sample of patients with a mean (SD) age of 79 (5.9) years, Kutchnia et al. [30] defined a new muscle quality indicator based on dual X-ray absorptiometry results for MM corrected according to ICW and ECW distributions calculated using BIA.

On the basis of the results of our study, we propose the ICW/LM ratio as assessed by BIA as an indicator of muscle quality, observing that sarcopenia defined using this quality criterion is related to physical performance indicators such as gait speed and TUG test results and to frailty in an expected, reasonable and plausible manner. Differences in the Barthel score did not acquire statistical significance probably because our study sample presented nearly optimal functional capacity at baseline (a mean Barthel score of 99.2) and because functional disability is probably a later consequence of sarcopenia. Poor muscle quality enables sarcopenia to be confirmed starting from possible sarcopenia established on the basis of an evaluation of muscle strength. In other words, combining the criteria of poor muscle quality and low muscle strength increases the ability to discriminate between people with poorer and better physical performance. Finally, we emphasize that the use of ICW/LM ratio as a muscle quality criterion in defining sarcopenia detects more cases of confirmed sarcopenia (compared to the former definition of sarcopenia based only on MM and strength) that would otherwise have gone undetected, thereby missing an opportunity for treatment. The greater number of cases of sarcopenia identified according to the quality criterion compared to the quantity (MM) criterion would suggest that muscle quality is lost before muscle quantity and is more related to loss of strength, corroborating recent studies [6,7,8,9].

Our study has some limitations. Firstly, body composition was assessed using a single-frequency non-segmental BIA device, which does not distinguish between lower and upper limb parameters. The formulas to estimate LM and ICW are not known because they are not made public by the device manufacturer. The Bodygram software provides numerous outputs, such as TBW, ECW, ICW and LM, all as indirect estimates derived from measures of resistance and reactance and other variables such as age, sex, weight, height and BMI. These estimates reflect the algorithms used to calculate the outputs. The algorithms differ from one BIA device to another because different inputs can be used [31]. Note, however, that the BIA device we use has been validated and is widely used in clinical research, including in aged populations, with a number of studies specifically confirming the accuracy of the ECW [32] and LM [33] estimates. Note also that LM and ICW estimates are not independent because most inputs considered are the same (although, without knowing the exact formula, possibly not all). If the inputs in both formulas are not exactly the same, different inputs add new information resulting in a non-perfect correlation between the variables. We observe a strong but not perfect correlation between ICW and LM: thus, for the same LM, ICW shows some variation between subjects. Most ICW variability depends on LM, but a small part of the variability may be due to cell volume or hydration. Finally, variability in the ICW/LM ratio does not seem to be due to errors in BIA conditions of use, since this ratio has been related with variables such as strength, gait speed and the TUG test in an expected and congruent way, which would tend to confirm the construct validity.

Thus, while muscle strength was assessed by hand grip, ICW content in LM was estimated for the whole body. This may explain the lack of an independent effect of the ICW/LM ratio on muscle strength. Secondly, the sample size, and especially the limited number of frail subjects, was such as to indicate a lack of power to detect statistically significant relationships in certain multivariate analyses and in the association between the ICW/LM ratio and frailty status. Finally, the study sample included a relatively homogeneous group of obese individuals aged 65–75 years with optimal functional capacity and the low variability in muscle strength, frailty status and functional status limited the study’s ability to detect possible associations with ICW content.

In conclusion, independently of age, sex and MM, ICW content in LM is related to certain physical performance parameters in obese people aged 65–75 years. Moreover, the use of the ICW/LM ratio as a muscle quality criterion in defining sarcopenia allows discrimination between subjects with different physical performance parameters and different frailty risk levels. Our study reinforces the idea that low-grade chronic cell dehydration may play a role in muscle function in aged populations, suggesting the need to pay more attention to hydration status in persons aged over 65 years. However, more studies are required to deepen our knowledge of the mechanisms through which age-related cell dehydration could be responsible for impaired muscle function and frailty.

## Figures and Tables

**Table 1:** Functional and body composition characteristics of the study sample.

	Total	Men	Women	*p*
Functional characteristics				
Barthel score	99.2 (6.0)	98.7 (9.9)	99.5 (1.7)	0.419
Gait speed (m/s)	1.01 (0.18)	1.08 (0.2)	0.98 (0.2)	<0.001
Timed up-and-go test (s)	10.2 (2.9)	9.6 (2.5)	10.6 (3.1)	0.001
Frailty status:				
Robust	118 (38.7%)	63 (60.6%)	55 (27.4%)	
Pre-frail	181 (59.3%)	41 (39.4%)	140 (69.7%)	<0.001
Frail	6 (2.0%)	0	6 (100%)	
Unipedal stance test (capable)	264 (86.6%)	93 (89.4%)	171 (85.1%)	0.291
Falls (in the last 3 months)	30 (9.8%)	11 (10.6%)	19 (9.5%)	0.755
Hand grip (kg)	22.1 (10.6)	33.1 (8.4)	16.4 (6.2)	<0.001
**Body composition**				
Weight (kg)	86.2 (11.2)	94.2 (9.6)	82.0 (9.6)	<0.001
Height (cm)	158.8 (8.8)	167.9 (5.4)	154.2 (6.2)	<0.001
TBW (mL)	40,433 (8092)	50,110 (49,800)	35,426 (36,655)	<0.001
TBW (as % of total weight)	46.8 (5.9)	53.4 (3.9)	43.4 (3.2)	<0.001
Extracellular water (% of TBW)	46.9 (5.6)	45.5 (6.1)	47.6 (5.2)	0.003
Intracellular water (% of TBW)	53.3 (6.3)	54.5 (6.1)	53.7 (6.3)	0.001
Fat mass (as % of total weight)	41.4 (7.6)	33.7 (5.9)	45.4 (4.9)	<0.001
Lean mass (as % of total weight)	58.5 (7.3)	66.7 (4.8)	54.2 (4.0)	<0.001
Muscle mass (as % of total weight)	38.0 (6.6)	44.2 (6.0)	34.8 (4.1)	<0.001

Numerical variables are expressed as mean (SD) and categorical variables as *n* (%). TBW: total body water.

**Table 2:** Relationship between the intracellular water content (ICW)/lean mass (LM) ratio and numerical clinical and functional characteristics.

−	r_s_ (*p*) *	β (*p*) ^†^
Age (years)	−0.086 (0.136)	−0.004 (0.193)
Body mass index	0.027 (0.640)	0.002 (0.518)
Number of comorbidities	−0.122 (0.032)	−0.003 (0.086)
Number of medications	−0.086 (0.136)	−0.008 (0.049)
Barthel score	−0.036 (0.536)	0.006 (0.355)
Gait speed (m/s)	0.355 (<0.001)	0.001 (<0.001)
Timed up-and-go test (s)	−0.452 (<0.001)	−0.017 (<0.001)
Hand grip (kg)	0.134 (0.020)	0.029 (0.015)
Men	0.171 (0.082)	0.020 (0.237)
Women	−0.060 (0.401)	0.002 (0.785)

* Results are expressed as Spearman’s correlation coefficients (r_s_). **^†^** Results are expressed as beta coefficients (*p*).

**Table 3:** Effect of the intracellular water/lean mass (ICW/LM) ratio on different functional outcomes (timed up-and-go (TUG) test, gait speed and hand grip) adjusted for age, sex and muscle mass (multivariate linear regression analyses).

Beta (*p*)	ICW/LM (mL/kg)	Age (years)	Female Sex	Muscle Mass (kg)	*R* ^2^
TUG test (s)	−0.015 (<0.001)	−0.008 (0.899)	0.520 (0.398)	−0.022 (0.603)	0.102
Gait speed (m/s)	0.001 (0.001)	0.000 (0.917)	−0.116 (0.002)	−0.001 (0.566)	0.124
Hand grip men (kg)	0.011 (0.656)	−0.556 (0.075)	---	0.042 (0.838)	0.047
Hand grip women (kg)	−0.007 (0.502)	−0.357 (0.026)	---	0.190 (0.103)	0.039

Results are expressed as beta coefficients (*p*).

**Table 4:** Effect of the intracellular water/lean mass (ICW/LM) ratio on balance and robustness adjusted for age, sex and muscle mass (multivariate logistic regression analyses).

	ICW/LM (mL/kg)	Age (years)	Female Sex	Muscle Mass (kg)	*R* ^2^
Able to stand on one foot	1.023 (1.01–1.04) *	0.840 (0.74–0.96) *	0.144 (0.04–0.58) *	0.865 (0.79–0.95) *	0.136
Robustness in men	1.011 (0.99–1.02)	0.962 (0.83–1.12)	---	0.968 (0.87–1.07)	0.053
Robustness in women	0.999 (0.99–1.01)	1.011 (0.90–1.13)	---	1.036 (0.95–1.13)	0.005

Results are expressed as odds ratio (95% confidence interval)/* *p* < 0.005.

**Table 5:** Comparison of frailty and physical performance indicators between subjects with possible sarcopenia and subjects with confirmed sarcopenia.

	Possible Sarcopenia (Low Strength)	Confirmed Sarcopenia (Low Strength + Poor Muscle Quality)	*p*
Frailty (%)	2.9 %	12.0%	0.085
Fallers (%)	8.6%	20.0%	0.098
Unipedal stance test (capable) (%)	82.9%	76.0%	0.405
Gait speed (m/s)	1.03 (0.17)	0.89 (0.20)	0.002
TUG (s)	9.74 (2.16)	12.64 (3.58)	<0.001
